# Supplementary material for: Analysis of culture and RNA isolation methods for precision-cut liver slices from cirrhotic rats
Source: Sci Rep. 2024 Jul 3;14:15349. doi: 10.1038/s41598-024-66235-2 (PMC11222550; doi:10.1038/s41598-024-66235-2)
Supplement: Supplementary file 1 — Supplementary Information. [file 41598_2024_66235_MOESM1_ESM.docx]

**Analysis of culture and RNA isolation methods for precision-cut liver slices from cirrhotic rats**

**Authors:** Ben D. Leaker, Yongtao Wang, Joshua Tam, R. Rox Anderson

**SUPPLEMENTAL INFORMATION**


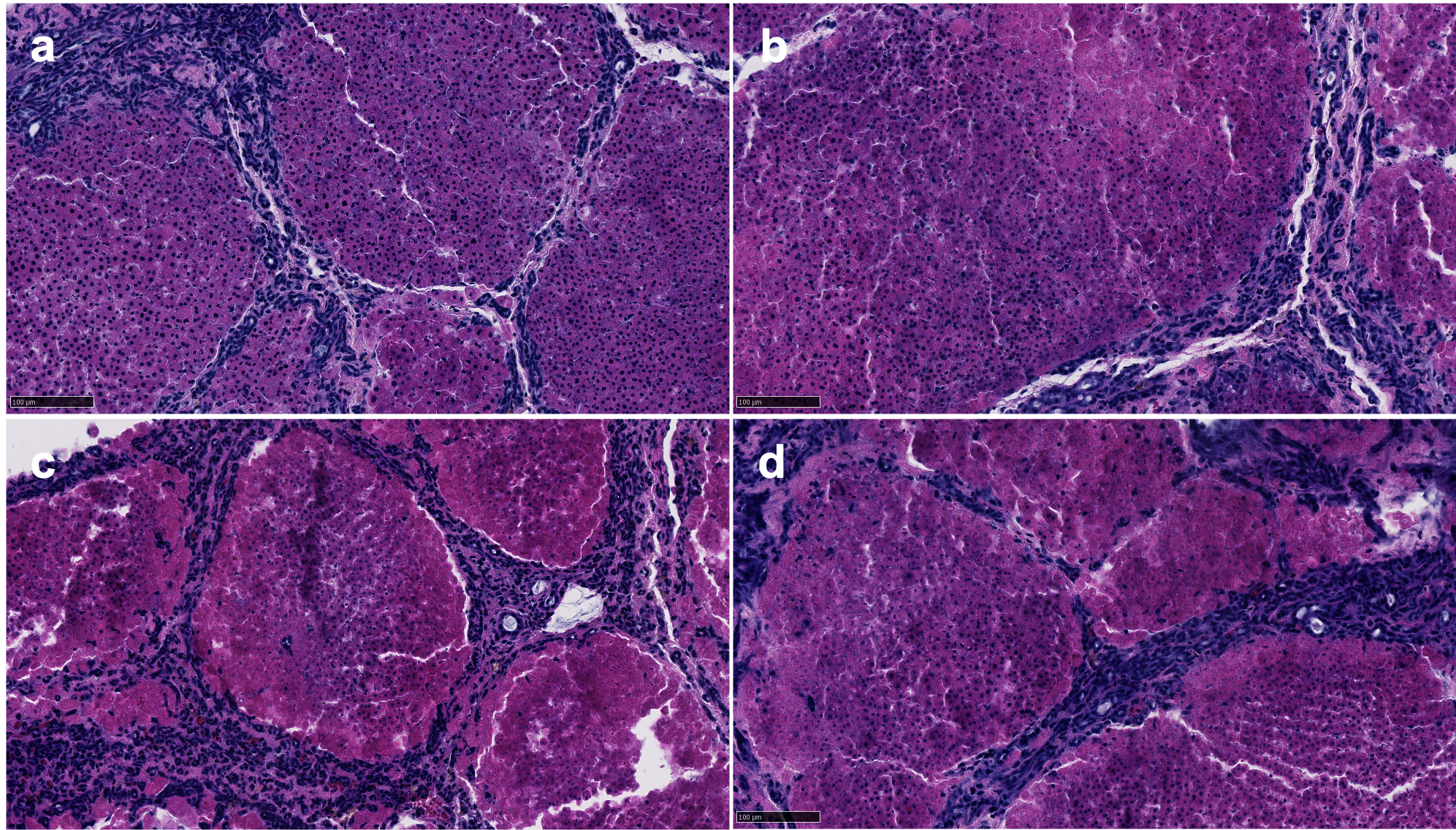


**Supplementary Figure 1: Representative H&E images for cirrhotic PCLS.** (a,b) Cirrhotic PCLS cultured for 2d and 4d, respectively, with insulin-transferrin-selenium-ethanolamine and dexamethasone on a rocking platform. Some small regions of necrosis develop between these timepoints. (c) Cirrhotic PCLS cultured for 4d without insulin-transferrin-selenium and dexamethasone on a rocking platform. Large regions of necrosis are evident. (d) Cirrhotic PCLS cultured for 4d with insulin-transferrin-selenium and dexamethasone without a rocking platform. Again, larger regions of necrosis have developed compared to PCLS cultured on a rocking platform. Scale bars are 100µm.


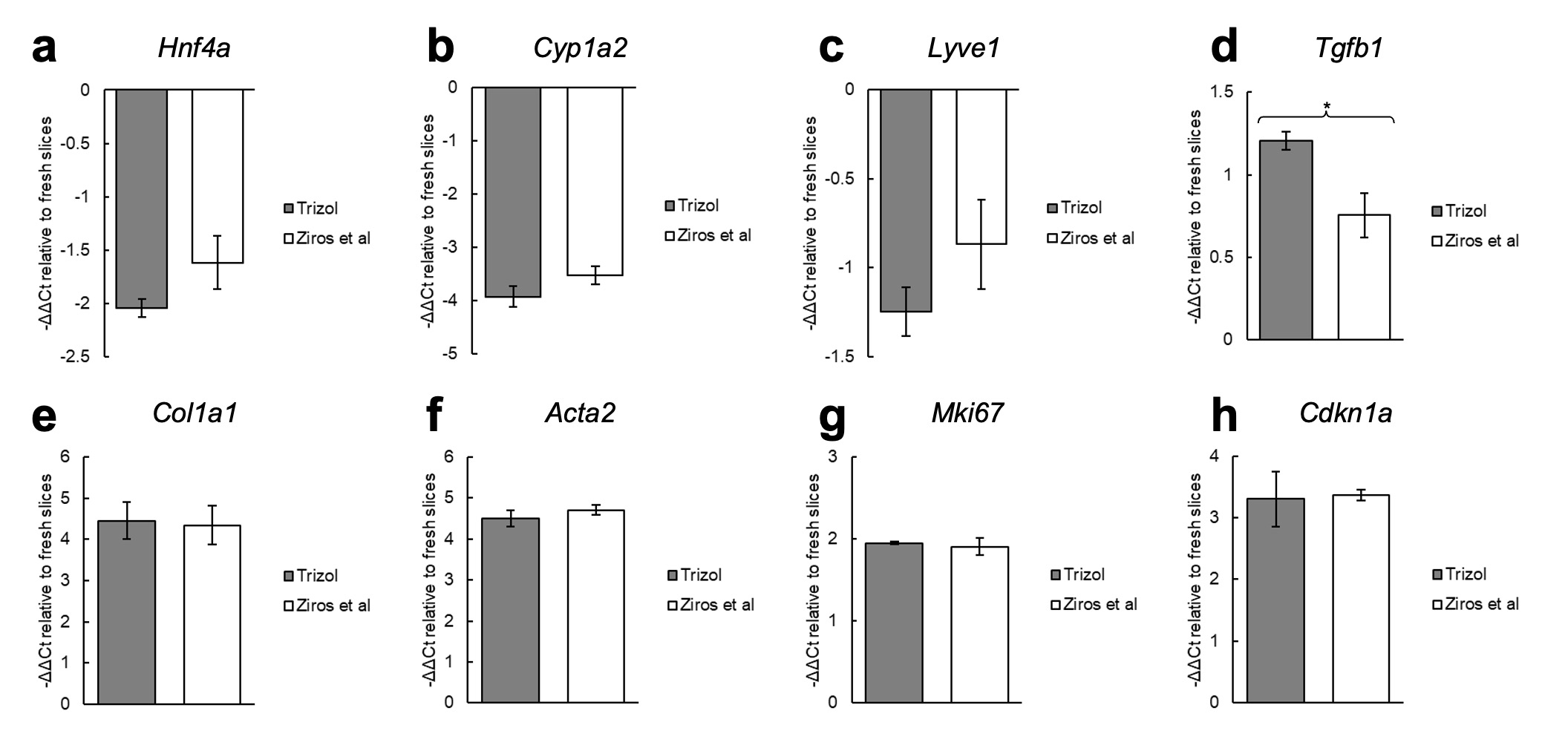


**Supplementary Figure 2: PCR results for RNA isolated with TRIzol protocol or Ziros et al. protocol from cirrhotic PCLS after 4 days in culture.** *p<0.05

| Time in Culture | Yield  (µg/mg tissue) | A$\frac{260}{280}$ | A$\frac{260}{230}$ |
| --- | --- | --- | --- |
| Fresh Slices | 6.3±1.8 | 2.08±0.02 | 2.1±0.1 |
| 4d | 2.3±0.9^*^ | 2.03±0.03^*^ | 1.9±0.4 |

**Supplementary Table 1: Yield and purity metrics for RNA isolated from fresh cirrhotic PCLS and after 4 days in culture.** RNA was isolated with the protocol described by Ziros et al. *p<0.05

| RNA Isolation  Protocol | Yield  (µg/mg tissue) | A$\frac{260}{280}$ | A$\frac{260}{230}$ |
| --- | --- | --- | --- |
| Qiagen RNeasy | 0.2±0.1 | 2.08±0.02 | 2.0±0.1 |
| TRIzol | 1.9±0.4 | 1.88±0.02 | 1.5±0.2 |
| Ziros et al. | 1.6±0.5* | 2.01±0.04*^†^ | 1.9±0.1^†^ |

**Supplementary Table 2: Yield and purity metrics for three methods of RNA isolation from healthy PCLS after 1 day in culture.** The protocol described by Ziros et al. gives the best combination of yield and RNA purity. *p<0.05 comparing Ziros et al. and Qiagen RNeasy protocols, ^†^p<0.05 comparing Ziros et al. and Trizol

| RNA Isolation  Protocol | Yield  (µg/mg tissue) | A$\frac{260}{280}$ | A$\frac{260}{230}$ |
| --- | --- | --- | --- |
| Qiagen RNeasy | 0.04±0.03 | 2.05±0.17 | 1.3±0.5 |
| TRIzol | 1.1±0.3 | 1.78±0.03 | 0.5±0.1 |
| Ziros et al. | 1.0±0.3* | 1.93±0.01^†^ | 1.6±0.1^†^ |

**Supplementary Table 3: Yield and purity metrics for three methods of RNA isolation from healthy PCLS after 4 days in culture.** The protocol described by Ziros et al. gives the best combination of yield and RNA purity. *p<0.05 comparing Ziros et al. and Qiagen RNeasy protocols, ^†^p<0.05 comparing Ziros et al. and Trizol
